# GWENA: gene co-expression networks analysis and extended modules characterization in a single Bioconductor package

**DOI:** 10.1186/s12859-021-04179-4

**Published:** 2021-05-25

**Authors:** Gwenaëlle G. Lemoine, Marie-Pier Scott-Boyer, Bathilde Ambroise, Olivier Périn, Arnaud Droit

**Affiliations:** 1 Département de médecine moléculaire, Faculté de médecine, Université Laval, 2325 rue de l’Université, Québec, G1V 0A6 Canada; 2 Centre de recherche du Chu de Quebec-Université Laval, 2705 boulevard Laurier Québec, Québec, G1V 4G2 Canada; 3 L’Oréal Research and Innovation, 15 rue Pierre Dreyfus, 92110 Clichy, France

**Keywords:** Co-expression network, Differential co-expression, R package, Pipeline, Aging, Skeletal muscle

## Abstract

**Background:**

Network-based analysis of gene expression through co-expression networks can be used to investigate modular relationships occurring between genes performing different biological functions. An extended description of each of the network modules is therefore a critical step to understand the underlying processes contributing to a disease or a phenotype. Biological integration, topology study and conditions comparison (e.g. wild vs mutant) are the main methods to do so, but to date no tool combines them all into a single pipeline.

**Results:**

Here we present GWENA, a new R package that integrates gene co-expression network construction and whole characterization of the detected modules through gene set enrichment, phenotypic association, hub genes detection, topological metric computation, and differential co-expression. To demonstrate its performance, we applied GWENA on two skeletal muscle datasets from young and old patients of GTEx study. Remarkably, we prioritized a gene whose involvement was unknown in the muscle development and growth. Moreover, new insights on the variations in patterns of co-expression were identified. The known phenomena of connectivity loss associated with aging was found coupled to a global reorganization of the relationships leading to expression of known aging related functions.

**Conclusion:**

GWENA is an R package available through Bioconductor (https://bioconductor.org/packages/release/bioc/html/GWENA.html) that has been developed to perform extended analysis of gene co-expression networks. Thanks to biological and topological information as well as differential co-expression, the package helps to dissect the role of genes relationships in diseases conditions or targeted phenotypes. GWENA goes beyond existing packages that perform co-expression analysis by including new tools to fully characterize modules, such as differential co-expression, additional enrichment databases, and network visualization.

**Supplementary Information:**

The online version contains supplementary material available at 10.1186/s12859-021-04179-4.

## Background

The study of biological functions through discrete genes analysis methods has allowed the elucidation of numerous pathways and the understanding of gene-disease associations [1]. The full comprehension of the complex interactions taking place in cellular processes requires methods that are able to grasp the connections between the genes involved [2]. To address this issue, biological networks have been used as a framework to represent and study relationships between genes. In a gene network, a node represents a gene and an edge joining two nodes represents their relationship. Among the measures of relationship, weighted co-expression is one of the most widely used thanks to the popularity of the WGCNA R package [3] where the relationships are quantified (weight) instead of only a presence/absence information. The use of gene co-expression networks thus led to important discoveries such as the characterization of functional elements in *Arabidopsis* [4], help with prognosis in breast cancer [5], and more generally identification and prioritization of disease candidate genes [6].

When constructing gene co-expression networks, existing tools usually follow the same methodology. Using either microarray or RNA-seq gene expression, a co-expression score based on correlation is computed between each pair of genes in the samples. A clustering method is then selected to detect groups of strongly co-expressed genes called modules. The search for meaning in the co-expression relations classically involves the integration of biological information, as well as the study of topology [6]. Biological integration usually involves two methods, namely gene set enrichment and phenotypic association [3, 6]. A phenotypic association is based on the correlation between the eigengene (a representative of gene expression profile) of the module and a phenotype measured on the samples. Despite typically having a low yet significant correlation [7], phenotypic associations are used as a surrogate to study the molecular changes related to a condition. By looking for the genes responsible for the correlation, this method serves as a means of causal genes discovery or a way to find the effect of the condition on the phenotype [8]. As for the gene set enrichment, the most common enrichment test is based on the over-representation analysis (ORA) of a group of genes (in this case modules) compared to a reference of biological annotations such as Gene Ontology (GO) [9] or Reactome [10]. This approach, based on the guilt-by-association approach, allows the identification of new gene functions. The consideration of the scale-free topology property of gene co-expression networks also allow the use of graph theory metrics and methods to analyze the networks from a new perspective. The highly-connected genes also known as hub genes are often relevant for the functionality of the module, either being a regulator [11] or a gene coding for an essential function [12]. Their detection and the investigation of the neighboring gene is therefore an opportunity to understand the mechanisms at work.

Like differential expression analysis, co-expression analysis can be used in a differential way to compare conditions (e.g. wild vs. mutant). This method aims to isolate dissimilarities [13] that would not be found by solely studying the GCN of a condition of interest (e.g. disease, phenotype). Variations in gene co-expression between multiple conditions can translate into appearance/disappearance of modules, changes in gene composition of a module, or rearrangement of genes within a module potentially leading to separation into several other modules [6]. These modifications of patterns reveal insights on the biological alterations in modules of interest and can suggest possible regulatory events linked to the studied condition (e.g.: transcription factors, miRNA). Such concepts were used successfully in recent publications to detect specific gene modules involved in ovarian or breast cancer [14, 15] or in recovery from water stress in *Cleistogenes* [16].

To date, multiple tools exist that perform one or few of the functionalities described previously but none combine them all into a single pipeline. Moreover, no available tool includes differential co-expression, exploits the potential of other topological metrics such as connectivity, or enables analysis to be carried out with other R packages or software as easily. In order to meet all these needs, we developed an R package for Gene Whole co-Expression Network Analysis (GWENA) available on Bioconductor (https://bioconductor.org/packages/release/bioc/html/GWENA.html). Based on a modified version of WGCNA for the network construction and module detection, GWENA is a modular pipeline that provides ORA enrichment on 9 biological sources, phenotypic association, hub genes detection, and differential co-expression between multiple conditions. These come with a set of descriptive visualizations that help the user understand and interpret complex results of gene co-expression network analysis.

In order to demonstrate the capabilities of our tool, we applied it to investigate skeletal muscle aging using publicly available gene expression data from donors spanning different age ranges from the GTEx database (ref). Skeletal muscle aging is indeed a major source of mobility loss in the elderly, resulting in a high fall ratio, depression, and therefore an increased mortality [17]. This decrease in the regenerative capacity of skeletal muscles and their progressive atrophy (sarcopenia) [18] gradually leads to a reduction of the contractile force and thus a loss of autonomy of the individuals [19]. Recent studies have made progress in finding factors associated to evolution of sarcopenia [17, 20], such as body weight [21], but the understanding of their intricate molecular mechanisms is still lacking.

In this article, we will therefore provide details on the implementation of our new R package GWENA. A presentation of its application will be done with the study of gene co-expression in young muscle, and then in the context of skeletal muscle aging by comparing samples from younger and older donors. Finally, a qualitative comparison will be made with other existing tools.

## Implementation

**Fig. 1:**
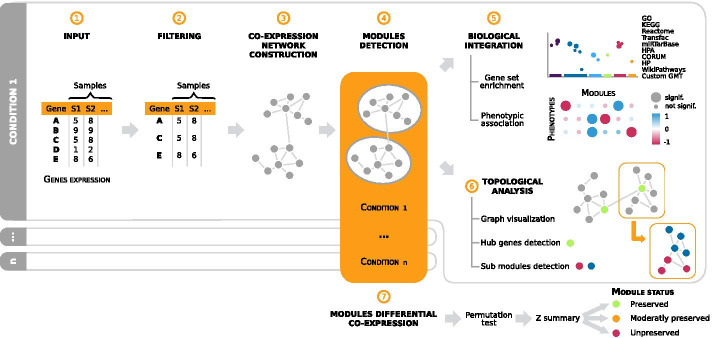
Detailed steps of analysis performed in GWENA’s pipeline, from expression data to characterization of the modules and comparison of conditions. ① Input : expression matrix pre-normalized and aggregated to gene level if it is a transcript matrix. ② Filtering : optional genes filtration according to transcriptomic input technology. ③ Co-expression network construction : computation through modified WGCNA function of a correlation matrix on the gene expression matrix, then transformation into an adjacency matrix, and finally into a topological overlap matrix (TOM). ④ Modules detection : genes clusterization over the TOM with another modified WGCNA function. ⑤ Biological integration : gene set enrichment of each module using g:Profiler services, and phenotypic association if a phenotype matrix is provided to describe the samples. ⑥ Graph analysis : transformation of the TOM in a graph to compute different topological metrics, detect the hub genes, and detect sub-modules of one module. ⑦ Modules differential co-expression over N conditions : permutation test using NetRep combined with a Z summary to detect preserved or unpreserved modules

Designed as an R Bioconductor package, GWENA is a modular pipeline intended to ease the construction, interpretation and comparison of GCN. It reproduces a classical GCN analysis reinforced by complementary tools (Fig. 1).

### Input

Both microarray and RNA-seq normalized expression can be used as input. The choice of normalization method is left to the user as it is highly dependent on the technology used to produce the raw data and the experimental design. Data must be stored in a table with genes as columns and samples as rows, or in a SummarizedExperiment object [22]. The minimal number of samples recommended is about 20 samples [23] with 100 samples ensuring a more robust networks [24].

Transcript-level data (probes or transcript) need to be aggregated to the gene level for the next steps (i.e. probes measurement summarized to their corresponding gene) [25]. Its execution is left to the user as the transcriptomic technology impacts the aggregation method to choose. However, it is recommended to use the highest mean probes expression for microarray data, and the counts sum for RNA-seq. This can be achieved with the collapsing R function as described by Miller et al. [25].

### Filtering

Genes are not always informative for modules detection as genes not always vary and their expression can be linked to technical biases. An additional filtering step can thus be applied to avoid noise and speed up the pipeline analysis. This operation must be carried out with caution as it may impact the network construction. Over-filtering may result in loss of informative signal and changing the data distribution could break the scale-free topology [23, 26]. In addition, co-expression network analysis is a method designed to handle larger amount of data than differential expression analyses and can capture more subtle significant gene expression variation [8, 27].

Two filters meeting these criteria are available in GWENA:

1. Low count filter : removes genes having a lower count than a pre-defined threshold (default is 5). It prevents confusing the true expression of a gene with an expression due to technical background noise.
2. Low variation filter : removes genes which expression is too similar across samples. As co-expression modules detection relies on the discrimination of similarity between gene expression profiles across samples, genes that do not vary sufficiently across samples may be randomly clustered in the same (or in different) modules which would not reflect the biological reality.

### Co-expression network construction

The well-known R package WGCNA [3] was modified in order to be integrated it in our modular pipeline : the co-expression network construction step which computes the genes pairwise co-expression score has been isolated in its own R function. The first step of the co-expression score computation is the calculation of a correlation matrix based on the gene expression matrix. The Spearman correlation was added to the automated version of network construction in WGCNA as it ensures a better representation of genes monotonic relationships [28]. A power law distribution is then fitted on the correlation matrix and the “correlation matrix is then raised to the estimated power, resulting in an adjacency matrix [29]. According to the hierarchical organization of gene co-expression networks [30], a topological overlap matrix (TOM) [29] is then computed using the adjacency matrix which represents the final gene co-expression score matrix. Finally, the function return this matrix along with metadata information regarding the computation to ensure a good tracking of the performed operations.

### Modules detection

The module detection part from WGCNA was isolated in a new R function using the previously calculated gene co-expression score matrix as input. A hierarchical clustering is performed on the matrix which is then cut according to the dynamic cut tree method [31] in order to define the modules and the genes they contain. The first component of the principal component analysis of each module is used as a representative of their respective gene expression profile and is called an eigengene. In addition to its summarizing function, the eigengene is used to merge the highly-correlated modules. The gene co-expression profile of each module is visible using a dedicated function, with the eigengene highlighted. The function finally returns a detailed object with the detected modules as lists of genes identifiers, the dendrogram of the clustering, and the modules before merge.

### Biological integration

Biological integration consists of two different analyses, namely gene set enrichment and phenotypic association.

The gene set enrichment (or functional enrichment) analysis is performed using g:Profiler [32] through their gprofiler2 R package. Their enrichment function covers 9 biological functional databases: Gene Ontology (GO) [9], Kyoto Encyclopedia of Genes and Genomes (KEGG) [33], Reactome [10], Transfac [34], miRTarBase [35], Human Protein Atlas (HPA) [36], CORUM [37], Human Phenotype ontology (HP) [38], WikiPathways [39]. Realizing a custom enrichment file through a Gene Matrix Transposed (GMT) format in gprofiler2 requires the use of additional functions. Also, gprofiler2 does not provide a merging function between the output of classical and custom enrichment to return all the enrichments in a single output. GWENA therefore provides a wrapper of these functions to have an all-in-one function.

The phenotypic association uses the eigengene returned in the output of the module detection function to perform a correlation test on a matrix of given phenotypes. If a phenotype is qualitative instead of quantitative, the variable encoding the phenotype is transformed into a binary variable (also known as dummy variable).

### Graph analysis

To analyze the topology of the graph and allow its visualization, GWENA imports the igraph [40] R package. A wrapping function including integrity checks use the gene co-expression score matrix to build a graph object on which all igraph topological metrics can be computed (e.g. degree, connectivity, strength). Among the multiple metrics computable on a network, hub genes remain the most studied structure. As they can be defined according to different methods, the three most popular ones were implemented: highest connectivity [41], highest degree [8], and Kleinberg’s score [42]. GWENA visualization function simplifies the native plotting function of igraph and adapts it to GCN to assist in their interpretation (e.g. the native implementation of an edge filter parameter, as these are complete graphs). The layout selection was also favored towards scale-free topology compatible layouts as they are a main property of GCN. Sub-modules within previously detected modules can provide valuable information about the distribution and communication between biological functions within a module. GWENA allows their detection by performing a partitioning around medoids (PAM) clustering method [43, 44] with an automatic estimation of the number of cluster through a silhouette coefficient. These sub-modules can also be passed to the graph plot function to display them and see their organization.

### Modules differential co-expression

Analysis of module preservation or non-preservation can be performed between different conditions such as treatments or phenotype. To isolate modules whose topology changes between conditions, GWENA first performs a permutation test using the NetRep R package [45]. Seven topological metrics are computed on each module in each condition. A permutation is then applied to the selected control condition where each node label of the modules is randomly reassigned without replacement to another and the seven metrics are then recomputed on it. Using these permutations as a null distribution [46], modules are considered preserved if all seven topological metrics are significant for the alternative hypothesis (one or two-sided) with the chosen alpha error.

As the unpreservation of a module cannot be assumed from the non-significant modules, a second step of preservation evaluation is carried out using a Z summary score [47, 48]. The final score returned by GWENA is the combination of these two steps (Additional file 1: Figure S1).

## Results and discussion

To present GWENA’s use and its capability to isolate genes groups or co-expression patterns of interest in a single condition or multiple conditions, we analyzed RNA-seq skeletal muscle data from GTEx (v8) [49] (Additional file 1: Table S1). This data set contains 19,312 genes from 803 samples representing ages ranging from 20 to 70 years old. Low read counts and the low variation genes were discarded using the filtering function of GWENA to decrease the noise, resulting in 18870 genes.

**Fig. 2:**
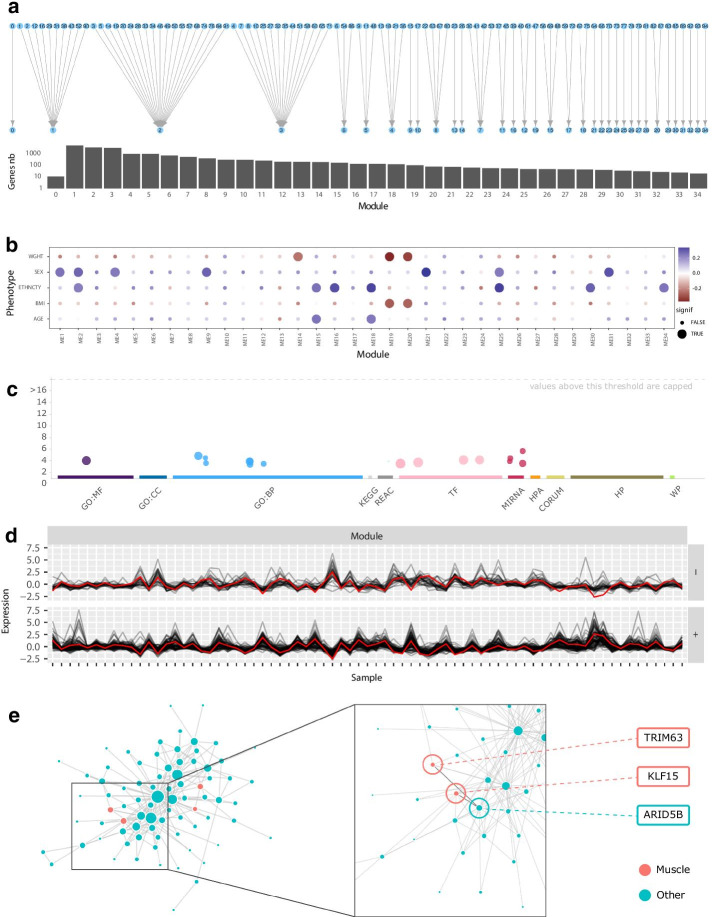
Available visualizations in GWENA along the pipeline applied to the aging study on the whole age range. **a** Modules merge as a bipartite graph from plot_modules_merge function and the genes distribution inside each of them (log scale). **b** Phenotypic association between the 35 modules and age, sex, BMI, ethnicity, weight. **c** Manhattan-like enrichment plot (interactive in GWENA) of module 10 on GO, KEGG, Reactome, Transfac, miRTarBase, Human Protein Atlas, CORUM, Human Phenotype ontology, WikiPathways. **d** Expression profile of module 19 split depending on the correlation sign to the eigengene. **e** Module 19’s network visualization as a graph with muscle enrichment genes colored in red and others in blue. The zoom focus on ENSG00000158022/ENSG00000107372/ENSG00000265972 and related hub genes

As GTEx data is known to be subject to multiple confounding factors (batch effect, experimental bias, read contamination, etc.) [26, 50, 51], a partial PC-correction [26] was applied to correct the data (Additional file 1: Figure S2 and S3). To investigate the aging process two subsets representing contrasting age classes were selected from the corrected data set: 73 samples between 20 and 30 years old (referred as young in this report), and 292 samples between 60 and 70 years old (referred as old in this report). Both datasets were analyzed using GWENA’s pipeline with default parameters, except for the correlation method parameter which was selected to be “spearman” instead of the default “pearson” as it is less prone to outliers.

**Fig. 3:**
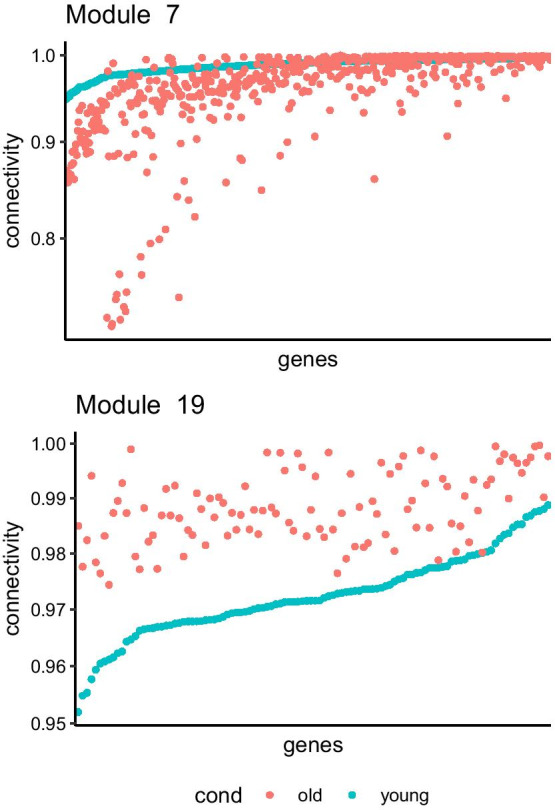
Modules 7 and 19 genes (nodes) connectivity distribution between young and old age ranges. Young age range is used as reference for sorting genes by increasing connectivity. A comparison over all modules can be found in Additional file 1: Figure S4

**Fig. 4:**
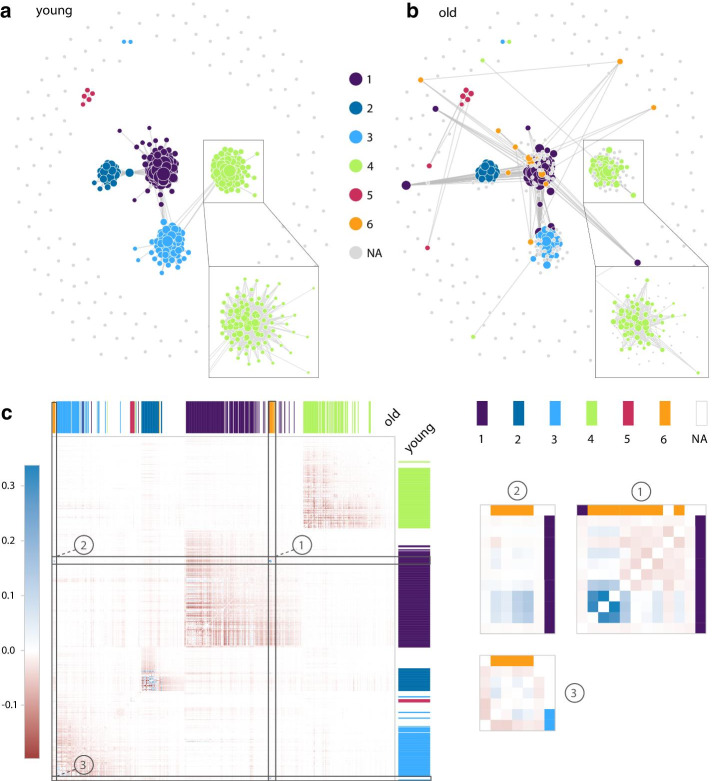
Module 7 network comparison between young and old. **a** Module 7 GCN graph plotted with GWENA (0.95 co-expression score filter) for young age range with sub-clusters detected. **b** Same as **a** but for the old age range. A zoom is made on sub-module 4 to show the peripheral genes disconnection. The new sub-module 6 is visible in purple in the old graph. **c** Difference network heatmap (old–young) ordered according to young age range network dendrogram. Sub-modules from old age range are visible on the top of the heatmap in columns, and sub-modules from young age range on the right in rows. Three zooms are made on the heatmap on the areas corresponding to sub-module 6 genes. Zoom ① contains the genes reconnecting in the old age range

**Table 1:** Summary of detected modules and related biological integration

Module	# genes	# pheno. asso.	# enrichment	% muscle enrich.
0	11	NA	NA	NA
1	5335	1	3288	0.6
2	3661	2	1098	0.4
3	3355	0	1620	0.3
4	1001	1	1883	1.0
5	987	0	1626	0.0
6	699	0	457	0.6
7	546	0	428	1.1
8	409	0	561	0.7
9	310	1	729	0.3
10	308	0	58	5.2
11	261	0	857	0.0
12	214	0	847	15.0
13	207	0	452	1.4
14	197	1	767	0.5
15	175	2	233	0.0
16	137	1	6	0.0
17	136	0	1	0.0
18	129	2	20	0.0
19	108	2	18	3.7
20	77	2	52	0.0
21	72	1	233	2.8
22	63	0	24	0.0
23	57	0	10	0.0
24	55	0	8	0.0
25	47	2	82	8.5
26	47	0	32	0.0
27	46	0	12	0.0
28	43	0	147	4.7
29	40	0	17	0.0
30	35	1	2	0.0
31	31	1	12	0.0
32	27	0	1	0.0
33	24	0	23	0.0
34	20	1	3	0.0

The number (#) of genes is indicated for each module (module 0 being a false module containing the unassigned genes. The number of phenotypic associations with the variables of interest (weight, sex, ethnicity, bmi, age) are counted for each one. The number of enrichments corresponds to the count of significant terms on each cumulative biological database. The ratio (%) of enriched terms associated with muscle is then established as the number of terms containing one or more elements of the following corpus: “muscle”, “sarco*”, “*”, “muscul*”, “actin*”, “myosin*” (where * denotes a completion by any other character string)

**Table 2:** Module 19 young enriched terms table

Source	Term name	*p* val.
GO:BP	Response to hormone	0.0015
GO:BP	Negative regulation of muscle hypertrophy	0.0033
GO:BP	Muscle adaptation	0.0118
GO:BP	Response to peptide hormone	0.0129
GO:BP	Striated muscle adaptation	0.0255
GO:BP	Platelet-derived growth factor receptor signaling pathway	0.0328
GO:BP	Regulation of muscle adaptation	0.0434
GO:MF	Enzyme binding	0.0097
MIRNA	hsa-miR-6882-5p	0.0002
MIRNA	hsa-miR-197-5p	0.0039
MIRNA	hsa-miR-152-5p	0.0125
MIRNA	hsa-miR-6878-5p	0.0282
REAC	Regulation of FOXO transcriptional activity by acetylation	0.0126
TF	Factor: Zbtb37; motif: NYACCGCRNTCACCGCR; match class: 1	0.0073
TF	Factor: RNF96; motif: BCCCGCRGCC; match class: 1	0.0074
TF	Factor: ETF; motif: GVGGMGG; match class: 1	0.0193
TF	Factor: AP-2; motif: SNNNCCNCAGGCN	0.0306
TF	Factor: AP-2; motif: SNNNCCNCAGGCN; match class: 0	0.0306

Multiple enrichment are linked to muscle development and growth

**Table 3:** Modules comparison between young and old age range and their comparison status

Comparison status	# modules	Modules id
Preserved	11	1, 2, 3, 4, 6, 8, 9, 11, 12, 14, 19
Moderately preserved	17	7, 10, 13, 17, 18, 20, 21, 22, 23, 24, 25, 27, 28, 30, 31, 33, 34
Unpreserved	2	16, 32
Inconclusive	4	5, 15, 26, 29

**Table 4:** Key features of GWENA compared to similar tools such as WGCNA, CEMiTool and wTO

**Functionnalities**	**Details**	**GWENA**	**WGCNA**	**CEMiTool**	**wTO**
Gene set enrichment	Gene ontology	yes	yes	no	no
	Pathways (KEGG/Reactome)	yes	no	no	no
	Regulation actors (TRANSFAC/miRTarBase)	yes	no	no	no
	Protein databases (Human Protein Atlas/CORUM)	yes	no	no	no
	Custom GMT import	yes	no	yes	no
Native network visualization		yes	no	no^a^	yes
Phenotype association		yes	yes	yes	no
Hub gene detection		yes	yes^b^	yes^c^	no
Igraph compatibility for extended topology metrics calculation		yes	no	no	no
Sub-module detections inside module & graph coloration accordingly		yes	no	no	no
Modules differential co-expression		yes	yes^d^	no	no^e^

As some differences remain under the same labels, details are provided about their content

^a^CEMiTool allows network visualization only if a protein-protein interaction network file is provided

^b^WGCNA only provides a single hub gene selection by module

^c^CEMiTool persistently provides the top 10 hub genes independently of the module size or connectivity

^d^WGCNA’s differential co-expression does not correct for multiple testing

^e^wTO have no differential co-expression method available but provides a consensus network method

### Single condition modules analysis

To illustrate the process of analyzing a single condition with GWENA, we initially focused on studying the muscle gene co-expression computed in the young sub-population. The 95 modules detected on the co-expression score matrix with GWENA were merged according to their similarity indices, which resulted in a total of 35 modules (Fig. 2a). Each module was then tested for its association with a selected set of phenotypes related to muscle aging (i.e. age, sex, ethnicity, body weight and BMI) to isolate modules of interest. As shown in Fig. 2b, 15 of these modules were significantly associated with at least one of the phenotypes.

These modules were provided to GWENA enrichment analysis (*p* value < 0.05 with g:SCS multiple testing correction) to identify their biological functions and assess their potential involvement in muscle function (Table 1). All modules were at least enriched in one term and 8 obtained enrichment terms related to muscle activity or metabolism (Table 1). Modules 19, 21 and 25 were the top 3 enriched for terms related to muscle function. However, modules 21 and 25 terms were mostly coming from Human Protein Atlas and were also related to a wide range of additional tissues such as the pancreas, the cervix, the bladder, the stomach, or the skin and were thus deemed less specific for muscle aging than module 19.

Briefly, the remaining module 19 presented 77% of genes positively correlated to its eigengene (therefore 23 negatively, Fig. 2d), and the muscle enriched terms involved muscle adaptation and negative regulation of hypertrophy (Table 2, Fig. 2c). The detection of hub genes by GWENA returned 12 hub genes, some of which are known as transcription factors. Among them, ARID5B (ENSG00000150347) is a transcription factor strongly co-expressed with KLF15 (ENSG00000163884) and TRIM63 (ENSG00000158022) (Fig. 2e). These two genes are present in the GO term GO:0014888 (striated muscle adaptation) to which ARID5B is not associated. The function of ARID5B is well known in adipocytes and hepatocytes but is still rarely studied in skeletal muscle metabolism. However, the knockout of this gene in mice has shown structural defects in the sarcomere structure [52].

Coupled with the results of GWENA, this may corroborate the involvement of ARID5B in the adaptation of striated muscle in response to a stimulus. Moreover, it has recently been shown that ARID5B knockout in mice was associated with increased glucose metabolism via an increased translocation of SLC2A4 (ENSG00000181856) [53]. Since SLC2A4 is a gene that is also regulated by KLF15 [54, 55], this supports the idea that ARID5B has implications in skeletal muscle function and more precisely in glucose metabolism. GWENA thus allowed the identification of a gene that may give new insight in the muscle development and growth which needs to be confirmed by further experiments.

### Multiple conditions modules comparison and analysis

Differential expression analysis allowed the detection of genes involved in aging in the last years (GenAge [56], Digital Ageing Atlas [57]). Such discriminant analysis is limited in helping to understand aging as this phenomenon is composed of concomitant mechanisms [58]. Understanding the relationships between the genes is therefore crucial to determine the altered functions and the changes involved. Differential GCN between conditions overcomes this problem by detecting the subtle pattern modifications. Using our previously defined young (20 to 30 years old) and old (50 to 60 years old) skeletal muscle modules, we ran GWENA’s GCN differential co-expression functionality to compare the modules between these age ranges. The GCN of each module detected in the young sub-population were taken as a reference and tested against the ones detected in the old sub-population.

From the 35 modules detected in the previously described single condition analysis of young muscle data, GWENA’s differential GCN of young versus old age range returned 2 modules that were unpreserved, 17 modules that were moderately preserved, 11 modules that were preserved, and 4 that were inconclusive (Table 3, Additional file 1: Figure S1). Unpreserved and moderately preserved modules are the most promising for identifying groups of genes differently expressed with age. Few and heterogeneous significant enrichment terms were associated to unpreserved modules while several moderately preserved modules had enrichment terms known to be linked to aging [58–61] such as transcription regulation (module 21), cellular stress (modules 20 and 27), immune response (modules 7 and 28), cell proliferation (module 13).

In addition to this biological information, the topological comparison of these modules allows to grasp the nature of the variations in the relationships between genes (nodes in the network) and their co-expression score (edge weight in the network). Connectivity, as defined by J. Dong and S. Horwath [62], is a common topological metric computed in GCN as it is representative of the network robustness and is known to be linked to network deregulation [63, 64]. Over all modules, the connectivity of the genes in module 7 was noticeably dropping between young and old age range (Fig. 3, Additional file 1: Figure S4). Using a co-expression score filter of 0.95, this loss of connectivity materialized in the network through a disconnection (edge loss) of peripheral genes (genes with low degree) such as in sub-module 4 between the young and old age range (Fig. 4a, b). Several other genes of the module 7 from the young age range also showed an increased connectivity when observed in the old age range, which therefore reflects a reconnection (edge gain) to other genes. These results confirm the observations from previous studies of a connectivity loss in the network of modules linked to aging [64, 65]. Overall, they support an alteration of the transcription regulation.

GWENA sub-module detection method on the module 7 revealed an impact of this reorganization of the gene connections by detecting 5 optimal sub-modules for the young age range, and 6 optimal sub-modules for the old age range. The gene composition of the sub-modules was highly similar between the condition (at least 81% common genes). Most of the differences in the gene composition are due to the disconnection of peripheral genes as previously spotted, and a small portion of the differences are due to the reconnection of genes or their attribution to another sub-module (Fig. 4a, b). This rewiring of the network is in line with known compensatory processes occurring during aging [60]. By triggering molecular processes involved in limiting or repairing cellular stress damage, these adaptive modifications aim to restore a homeostatic state.

To support this information, the new sub-module (sub-module 6 in Fig. 4b) appearing in the old age range was investigated further. Its creation is at the expense of the sub-module 1 of the young age range and of the 13 genes composing it, 8 of the genes are from the sub-module 1, 3 are reconnecting genes, and 2 are from sub-modules 2 and 3. A gene set enrichment analysis with GWENA of this sub-module 6 revealed significant enrichment in functions related to wound healing, coagulation, vessel diameter, platelet degranulation, and plasminogen activation (Additional file 1: Table S2). This is coherent with known morphological alterations of the vascular system in the aged skeletal muscle [66, 67], and the global immune/inflammatory response increased in aging [61]. Also, 51 enrichments from young sub-module 1 were not found significant in any of the sub-modules in the old age range. These enrichments involve antibacterial humoral response and negative regulation of endopeptidase activity. These terms are known to be associated with satellite cells (muscle stem cells responsible for muscle regeneration) regulators released by the vasculature in higher quantity in young skeletal muscle [67].

To complement these analyses, we investigated the variations of co-expression scores leading to the appearance of sub-module 6 in the old age range. Using the network co-expression matrix $\delta$ returned by GWENA for each condition, a co-expression difference matrix (Fig. 4c) was computed such as $\delta _{old} - \delta _{young}$. In this matrix, gene pairs with a negative score indicates a decrease in the co-expression over aging while a positive score indicates an increase in the co-expression. Among the variations, 3 genes showed a significant increase in co-expression between them but also towards other genes of sub-module 6. The pattern visible in Fig. 4c ① and ② suggest that these genes may be driving the co-expression changes occurring in this sub-module. These genes are FGG (ENSG00000171557), FGA (ENSG00000171560), and FGB (ENSG00000171564), the three fibrinogen chain coding genes involved in the polymerization of a fibrin matrix. This finding is consistent with previous studies about the increasing fibrinogen content in the elderly skeletal muscle leading to persistent fibrin deposition preventing myofiber repair [68, 69]. They also support the hypothesis of an inflammatory response triggered by a fibrin accumulation. All these results allowed by GWENA’s capacity to study the topology and the biological context easily tend to support the idea of not only a global loss in connectivity in aging but also of a gene co-expression reorganization. Our tools also highlighted the biological translation of this reorganization as a potential compensatory response from antibacterial humoral response and endopeptidase activity towards coagulation and wound response.

### GWENA’s contribution and comparison with existing tools

Weighted GCN can be computed from existing tools such as WGCNA [3], wTO [70], CEMiTool [71]. As both GWENA and CEMiTool use elements from WGCNA, they share notable functionalities. They use similar network construction and modules detection functions from WGCNA but offer their own filter on the datasets. GWENA has been enhanced with additional checks on the network construction (such as aberrant power check) compared to WGCNA and CEMiTool. On its side, wTO used a different version of a topological score to construct the network as they don’t perform a power law conversion on the correlation matrix and don’t use the same definition of topological transformation. Therefore, the main differences between WGCNA, wTO, CEMiTool and GWENA lie in the added functionalities for module analysis.

Regarding biological integration, wTO provides neither phenotypic association nor gene set enrichment. The other three tools allow phenotypic association but differ on gene set enrichment analysis. While CEMiTool only allows enrichment on imported GMTs, WGCNA and GWENA allow enrichment on gene ontology. GWENA is the only one allowing enrichment on other databases of pathways, regulatory agents, and proteins (in addition to imported GMTs).

Additional topological analysis functions are also available in several of these tools. The most common, hub gene detection, is present in WGCNA, CEMiTool, and GWENA in different forms. CEMitool and WGCNA offer respectively as hub gene the top 10 most connected genes and genes with a top kME score (membership module based on eigengene). However, methods based on a fixed number of hub genes tend to bias the information since the number of hub genes can vary according to the number of genes present in the module. GWENA therefore proposes several methods (highest connectivity, superior degree, Kleinberg’s score) based on a selection of genes with a hub score above a threshold. Another addition specific to GWENA is the ability to re-detect sub-modules into a defined module in order to further investigate the co-expression reconnection organization, and then identify the relations between enrichments associated to each sub-module by visualizing them on the graph plot.

GWENA includes a differential co-expression analysis in the analysis pipeline as opposed to packages dedicated solely to it (DiffCoEx [72], CoDiNA [73], CoXpress [74]) or packages like wTO or CEMiTool that do not contain this analysis. The method in GWENA differs from the one present in WGCNA in that it includes a permutation test to prevent the problem of multi-testing. With the addition of the Z-summary score to detect unpreserved modules, GWENA is therefore the only pipeline including a differential co-expression analysis with high confidence in modules found unpreserved. The Table 4 finally provide a head-to-head comparison for all functionalities made available by GWENA.

CEMiTool and wTO are built as stand-alone tools with little or no eased interfacing with other tools. WGCNA is similar to them except for exporting networks to Cytoscape [75] or VisANT [76]. Conversely, GWENA has been developed according to a modular architecture in order to facilitate the realization with an external tool of one of the stages of the analysis pipeline defined in Fig. 1. GWENA will thus be more easily adaptable to follow future developments in co-expression network analysis methodology.

GWENA, as other GCN analysis tool, has limitations. A first one common to all GCN construction method is that the quality of input data (e.g. filtration and/or proper normalization) will inevitably bias the results, especially if it breaks the scale-free property. A second limitation is the design of the permutation test that prevents reporting a significant unpreservation. The non-rejection of the null hypothesis of unpreservation can only state a lack of evidence of preservation. Therefore, unpreserved modules are determined among these modules lacking evidence (the non-significant modules) by the calculation of Z summary which only provide a tendency in the unpreservation [47]. The present application of GWENA to skeletal muscle aging also presents its own limitation. All analyses were performed on skeletal muscle sample and results were commented regarding this context. However, to be sure of the specificity of the findings, an additional differential co-expression of the modules should be performed on samples from other tissues from subjects with similar age range. As single-cell technologies are becoming common, the differential co-expression could also be used to target the cell-to-cell specific aging variation inside a tissue. Finally, as co-expression networks were unsigned and aging is a complex phenomenon involving actors beyond gene expression, causal effect of any finding need to be experimentally verified.

## Conclusion

In this paper, we introduced GWENA, an R package on Bioconductor to construct and analyze GCN in a single pipeline through a whole range of tools from biological integration, topological analysis, and differential co-expression. The package reduces complexity of the GCN analysis through simple input and output functions combined to a set of visualizations to explore the results. The separation of each step of the analysis in one function also allows quick and easy replacement if users wish to use another method for this block.

GWENA demonstrated its performances on both single and multiple condition analysis through an exploration of variations of skeletal muscle function and processes in aging. The single condition analysis showed it is possible to find new genes potentially involved in an existing GO annotation using hub genes, network neighboring genes and gene sets enrichments. The differential co-expression analysis between young and old samples isolated modules specifically linked to aging and detected the rearrangement in connectivity related to aging. Additional analysis supported the observed genes co-expression reorganization beyond simple connectivity loss. This resulted in a reinforcement of previous supposition on inflammatory response to fibrin increases in skeletal muscle aging.

## Supplementary Information

**Additional file 1.** **Supplementary Figure 4:** Distribution of the connectivity for each gene by module between the two age range. Genes connectivity is ordered by increasing connectivity in the young condition (red).

## Data Availability

Project name : Gene Whole co-Expression Network Analysis (GWENA) Operating system(s): Platform independent Programming language: R Package on Bioconductor : https://bioconductor.org/packages/devel/bioc/html/GWENA.html Package GitHub repository (development, issues, and pull requests) : https://github.com/Kumquatum/GWENA. Licence : GPL-3 GTEx v8 public data (RNA-seq and anonymized phenotype) : https://gtexportal.org/home/datasets GTEx detailed phenotype requires a dbGaP request.

## Abbreviations

GWENA: Gene Whole co-Expression Network Analysis
GCN: Gene Co-expression Network
GO: Gene Ontology
GO:MF: GO Molecular Function
GO:BP: GO Biological Process
GO:CC: GO Cellular Compartment
KEGG: Kyoto Encyclopedia of Genes and Genomes
REAC: Reactome
WP: WikiPathway
TF: TRANSFAC
MIRNA: miRTarBase
HPA: Human Protein Atlas
CORUM: Comprehensive Resource of Mammalian protein complexes
HP: Human Phenotype Ontology
GMT: Gene Matrix Transposed file format
DE: Differential Expression
TOM: Topological Overlap Matrix
ORA: Over Representation Analysis
BMI: Body Mass Index
ENSG: Ensembl Gene ID (human)

## References

[1] Barabási AL, Oltvai ZN (2004). Network biology: understanding the cell’s functional organization. Nat Rev Genet.

[2] Hartwell LH, Hopfield JJ, Leibler S, Murray AW (1999). From molecular to modular cell biology. Nature.

[3] Langfelder P, Horvath S (2008). WGCNA: an R package for weighted correlation network analysis. BMC Bioinform.

[4] Mao L, Van Hemert JL, Dash S, Dickerson JA (2009). Arabidopsis gene co-expression network and its functional modules. BMC Bioinform.

[5] Tang J, Kong D, Cui Q, Wang K, Zhang D, Gong Y, Wu G (2018). Prognostic genes of breast cancer identified by gene co-expression network analysis. Front Oncol.

[6] van Dam S, Võsa U, van der Graaf A, Franke L, de Magalhães JP (2017). Gene co-expression analysis for functional classification and gene-disease predictions. Brief Bioinform.

[7] Zhang B, Gaiteri C, Bodea LG, Wang Z, McElwee J, Podtelezhnikov AA, Zhang C, Xie T, Tran L, Dobrin R, Fluder E, Clurman B, Melquist S, Narayanan M, Suver C, Shah H, Mahajan M, Gillis T, Mysore J, MacDonald ME, Lamb JR, Bennett DA, Molony C, Stone DJ, Gudnason V, Myers AJ, Schadt EE, Neumann H, Zhu J, Emilsson V (2013). Integrated systems approach identifies genetic nodes and networks in late-onset Alzheimer’s disease. Cell.

[8] Tseng GC, Sibille E, Gaiteri C, Ding Y, French B (2013). Beyond modules and hubs: the potential of gene coexpression networks for investigating molecular mechanisms of complex brain disorders. Genes Brain Behav.

[9] Ashburner M, Ball CA, Blake JA, Botstein D, Butler H, Cherry JM, Davis AP, Dolinski K, Dwight SS, Eppig JT, Harris MA, Hill DP, Issel-Tarver L, Kasarskis A, Lewis S, Matese JC, Richardson JE, Ringwald M, Rubin GM, Sherlock G (2000). Gene ontology: tool for the unification of biology. Nat Genet.

[10] Fabregat A, Sidiropoulos K, Garapati P, Gillespie M, Hausmann K, Haw R, Jassal B, Jupe S, Korninger F, McKay S, Matthews L, May B, Milacic M, Rothfels K, Shamovsky V, Webber M, Weiser J, Williams M, Wu G, Stein L, Hermjakob H, D’Eustachio P (2016). The reactome pathway knowledgebase. Nucleic Acids Res.

[11] Pierson E, Koller D, Battle A, Mostafavi S (2015). Sharing and specificity of co-expression networks across 35 human tissues. PLoS Comput Biol.

[12] Hahn MW, Kern AD (2005). Comparative genomics of centrality and essentiality in three eukaryotic protein-interaction networks. Mol Biol Evol.

[13] Chowdhury HA, Bhattacharyya DK, Kalita JK (2019). (Differential) Co-expression analysis of gene expression: a survey of best practices. IEEE/ACM Trans Comput Biol Bioinform.

[14] Gov E, Arga KY (2017). Differential co-expression analysis reveals a novel prognostic gene module in ovarian cancer. Sci Rep.

[15] Bhuva DD, Cursons J, Smyth GK, Davis MJ (2019). Differential co-expression-based detection of conditional relationships in transcriptional data: comparative analysis and application to breast cancer. Genome Biol.

[16] Yan Q, Wu F, Yan Z, Li J, Ma T, Zhang Y, Zhao Y, Wang Y, Zhang J (2019). Differential co-expression networks of long non-coding RNAs and mRNAs in *Cleistogenes songorica* under water stress and during recovery. BMC Plant Biol.

[17] Bulut EA, Soysal P, Aydin AE, Dokuzlar O, Kocyigit SE, Isik AT (2017). Vitamin B12 deficiency might be related to sarcopenia in older adults. Exp Gerontol.

[18] Santilli V, Bernetti A, Mangone M, Paoloni M (2014). Clinical definition of sarcopenia. Clin Cases Miner Bone Metab.

[19] Janssen I, Heymsfield SB, Ross R (2002). Low relative skeletal muscle mass (sarcopenia) in older persons is associated with functional impairment and physical disability. J Am Geriatr Soc.

[20] Sakuma K, Aoi W, Yamaguchi A (2017). Molecular mechanism of sarcopenia and cachexia: recent research advances. Pflugers Arch.

[21] Jiao J, Demontis F (2017). Skeletal muscle autophagy and its role in sarcopenia and organismal aging. Curr Opin Pharmacol.

[22] Morgan M, Obenchain V, Hester J, Pagès, H. SummarizedExperiment: Summarized-Experiment container, 2018 (2018)

[23] Langfelder PHS. Frequently asked questions. 2014. https://horvath.genetics.ucla.edu/html/CoexpressionNetwork/Rpackages/WGCNA/faq.html. Accessed 26 Aug 2020.

[24] Liesecke F, De Craene JO, Besseau S, Courdavault V, Clastre M, Vergès V, Papon N, Giglioli-Guivarc’h N, Glévarec G, Pichon O, Dugé-Bernonville T (2019). Improved gene co-expression network quality through expression dataset down-sampling and network aggregation. Sci Rep.

[25] Miller JA, Cai C, Langfelder P, Geschwind DH, Kurian SM, Salomon DR, Horvath S (2011). Strategies for aggregating gene expression data: the collapserows R function. BMC Bioinform.

[26] Parsana P, Ruberman C, Jaffe AE, Schatz MC, Battle A, Leek JT (2019). Addressing confounding artifacts in reconstruction of gene co-expression networks. Genome Biol.

[27] Hudson NJ, Reverter A, Dalrymple BP (2009). A differential wiring analysis of expression data correctly identifies the gene containing the causal mutation. PLoS Comput Biol.

[28] Song L, Langfelder P, Horvath S (2012). Comparison of co-expression measures: mutual information, correlation, and model based indices. BMC Bioinform.

[29] Yip AM, Horvath S (2007). Gene network interconnectedness and the generalized topological overlap measure. BMC Bioinform.

[30] Ravasz E, Barabási AL (2003). Hierarchical organization in complex networks. Phys Rev E Stat Phys Plasmas Fluids Relat Interdiscip Top.

[31] Langfelder P, Zhang B, Horvath S (2008). Defining clusters from a hierarchical cluster tree: the dynamic tree cut package for R. Bioinformatics.

[32] Raudvere U, Kolberg L, Kuzmin I, Arak T, Adler P, Peterson H, Vilo J (2019). g:Profiler: a web server for functional enrichment analysis and conversions of gene lists (2019 update). Nucleic Acids Res.

[33] Kanehisa M, Sato Y, Furumichi M, Morishima K, Tanabe M (2019). New approach for understanding genome variations in KEGG. Nucleic Acids Res.

[34] Matys V, Kel-Margoulis OV. Fricke E, Liebich I, Land S, Barre-Dirrie A, Reuter I, Chekmenev D, Krull M, Hornischer K, Voss N, Stegmaier P, Lewicki-Potapov B, Saxel H, Kel AE, Wingender E. TRANSFAC and its module TRANSCompel: transcriptional gene regulation in eukaryotes. Nucleic Acids Res. 2006;34(Database issue):108–10. 10.1093/nar/gkj143. 10.1093/nar/gkj143 PMC1347505 16381825

[35] Chou CH, Shrestha S, Yang CD, Chang NW, Lin YL, Liao KW, Huang WC, Sun TH, Tu SJ, Lee WH, Chiew MY, Tai CS, Wei TY, Tsai TR, Huang HT, Wang CY, Wu HY, Ho SY, Chen PR, Chuang CH, Hsieh PJ, Wu YS, Chen WL, Li MJ, Wu YC, Huang XY, Ng FL, Buddhakosai W, Huang PC, Lan KC, Huang CY, Weng SL, Cheng YN, Liang C, Hsu WL, Huang HD. MiRTarBase update 2018: a resource for experimentally validated microRNA-target interactions. Nucleic Acids Res. 2018;46(D1):296–302. 10.1093/nar/gkx1067. 10.1093/nar/gkx1067 PMC5753222 29126174

[36] Uhlen M, Fagerberg L, Hallstrom BM, Lindskog C, Oksvold P, Mardinoglu A, Sivertsson A, Kampf C, Sjostedt E, Asplund A, Olsson I, Edlund K, Lundberg E, Navani S, Szigyarto CA-K, Odeberg J, Djureinovic D, Takanen JO, Hober S, Alm T, Edqvist P-H, Berling H, Tegel H, Mulder J, Rockberg J, Nilsson P, Schwenk JM, Hamsten M, von Feilitzen K, Forsberg M, Persson L, Johansson F, Zwahlen M, von Heijne G, Nielsen J, Ponten F (2015). Tissue-based map of the human proteome. Science.

[37] Ruepp A, Brauner B, Dunger-Kaltenbach I, Frishman G, Montrone C, Stransky M, Waegele B, Schmidt T, Doudieu ON, Stümpflen V, Mewes HW (2008). CORUM: the comprehensive resource of mammalian protein complexes. Nucleic Acids Res.

[38] Köhler S, Carmody L, Vasilevsky N, Jacobsen JOB, Danis D, Gourdine JP, Gargano M, Harris NL, Matentzoglu N, McMurry JA, Osumi-Sutherland D, Cipriani V, Balhoff JP, Conlin T, Blau H, Baynam G, Palmer R, Gratian D, Dawkins H, Segal M, Jansen AC, Muaz A, Chang WH, Bergerson J, Laulederkind SJF, Yüksel Z, Beltran S, Freeman AF, Sergouniotis PI, Durkin D, Storm AL, Hanauer M, Brudno M, Bello SM, Sincan M, Rageth K, Wheeler MT, Oegema R, Lourghi H, Della Rocca MG, Thompson R, Castellanos F, Priest J, Cunningham-Rundles C, Hegde A, Lovering RC, Hajek C, Olry A, Notarangelo L, Similuk M, Zhang XA, Gómez-Andrés D, Lochmüller H, Dollfus H, Rosenzweig S, Marwaha S, Rath A, Sullivan K, Smith C, Milner JD, Leroux D, Boerkoel CF, Klion A, Carter MC, Groza T, Smedley D, Haendel MA, Mungall C, Robinson PN (2019). Expansion of the Human Phenotype Ontology (HPO) knowledge base and resources. Nucleic Acids Res.

[39] Slenter DN, Kutmon M, Hanspers K, Riutta A, Windsor J, Nunes N, Mélius J, Cirillo E, Coort SL, DIgles D, Ehrhart F, Giesbertz P, Kalafati M, Martens M, Miller R, Nishida K, Rieswijk L, Waagmeester A, Eijssen LMT, Evelo CT, Pico AR, Willighagen EL. WikiPathways: a multifaceted pathway database bridging metabolomics to other omics research. Nucleic Acids Res. 46(D1):661–667 (2018). 10.1093/nar/gkx1064. 10.1093/nar/gkx1064 PMC5753270 29136241

[40] Gabor C, Tamas N (2006). The igraph software package for complex network research. InterJ Complex Syst.

[41] Azuaje FJ (2014). Selecting biologically informative genes in co-expression networks with a centrality score. Biol Direct.

[42] Kleinberg JM (1999). Authoritative sources in a hyperlinked environment. J ACM.

[43] Kaufmann L, Rousseeuw P. Clustering by means of medoids. Data analysis based on the L1-norm and related methods; 1987. p. 405–16.

[44] Schubert E, Rousseeuw PJ, Amato G, Gennaro C, Oria V, Miloš R (2019). Faster k-medoids clustering: improving the PAM, CLARA, and CLARANS algorithms. Similarity search and applications.

[45] Ritchie SC, Watts S, Fearnley LG, Holt KE, Abraham G, Inouye M (2016). A scalable permutation approach reveals replication and preservation patterns of network modules in large datasets. Cell Syst.

[46] Phipson B, Smyth GK. Permutation p-values should never be zero: calculating exact p-values when permutations are randomly drawn. Stat Appl Genet Mol Biol. 2010;9(1). 10.2202/1544-6115.1585 21044043

[47] Langfelder P, Luo R, Oldham MC, Horvath S. Is my network module preserved and reproducible? PLoS Comput Biol. 2011;7(1). 10.1371/journal.pcbi.1001057 10.1371/journal.pcbi.1001057 PMC3024255 21283776

[48] Li B, Zhang Y, Yu Y, Wang P, Wang Y, Wang Z, Wang Y (2015). Quantitative assessment of gene expression network module-validation methods. Sci Rep.

[49] Ardlie KG, DeLuca DS, Segrè AV, Sullivan TJ, Young TR, Gelfand ET, Trowbridge CA, Maller JB, Tukiainen T, Lek M, Ward LD, Kheradpour P, Iriarte B, Meng Y, Palmer CD, Esko T, Winckler W, Hirschhorn JN, Kellis M, MacArthur DG, Getz G, Shabalin AA, Li G, Zhou YH, Nobel AB, Rusyn I, Wright FA, Lappalainen T, Ferreira PG, Ongen H, Rivas MA, Battle A, Mostafavi S, Monlong J, Sammeth M, Melé M, Reverter F, Goldmann JM, Koller D, Guigó R, McCarthy MI, Dermitzakis ET, Gamazon ER, Im HK, Konkashbaev A, Nicolae DL, Cox NJ, Flutre T, Wen X, Stephens M, Pritchard JK, Tu Z, Zhang B, Huang T, Long Q, Lin L, Yang J, Zhu J, Liu J, Brown A, Mestichelli B, Tidwell D, Lo E, Salvatore M, Shad S, Thomas JA, Lonsdale JT, Moser MT, Gillard BM, Karasik E, Ramsey K, Choi C, Foster BA, Syron J, Fleming J, Magazine H, Hasz R, Walters GD, Bridge JP, Miklos M, Sullivan S, Barker LK, Traino HM, Mosavel M, Siminoff LA, Valley DR, Rohrer DC, Jewell SD, Branton PA, Sobin LH, Barcus M, Qi L, McLean J, Hariharan P, Um KS, Wu S, Tabor D, Shive C, Smith AM, Buia SA, Undale AH, Robinson KL, Roche N, Valentino KM, Britton A, Burges R, Bradbury D, Hambright KW, Seleski J, Korzeniewski GE, Erickson K, Marcus Y, Tejada J, Taherian M, Lu C, Basile M, Mash DC, Volpi S, Struewing JP, Temple GF, Boyer J, Colantuoni D, Little R, Koester S, Carithers LJ, Moore HM, Guan P, Compton C, Sawyer SJ, Demchok JP, Vaught JB, Rabiner CA (2015). Lockhart: The Genotype-Tissue Expression (GTEx) pilot analysis: multitissue gene regulation in humans. Science.

[50] Nieuwenhuis TO, Yang SY, Verma RX, Pillalamarri V, Arking DE, Rosenberg AZ, McCall MN, Halushka MK (2020). Consistent RNA sequencing contamination in GTEx and other data sets. Nat Commun.

[51] Somekh J, Shen-Orr SS, Kohane IS (2019). Batch correction evaluation framework using a-priori gene-gene associations: applied to the GTEx dataset. BMC Bioinform.

[52] Murray J, Whitson RH, Itakura K (2018). Reduced prostaglandin I2 signaling in Arid5b2/2 primary skeletal muscle cells attenuates myogenesis. FASEB J.

[53] Okazaki Y, Murray J, Ehsani A, Clark J, Whitson RH, Hirose L, Yanaka N, Itakura K (2020). Increased glucose metabolism in Arid5b -/- skeletal muscle is associated with the down-regulation of TBC1 domain family member 1 (TBC1D1). Biol Res.

[54] Gray S, Feinberg MW, Hull S, Kuo CT, Watanabe M, Sen S, Depina A, Haspel R, Jain MK (2002). The Krüppel-like factor KLF15 regulates the insulin-sensitive glucose transporter GLUT4. J Biol Chem.

[55] Fan L, Hsieh PN, Sweet DR, Jain MK (2018). Krüppel-like factor 15: regulator of BCAA metabolism and circadian protein rhythmicity. Pharmacol Res.

[56] Tacutu R, Thornton D, Johnson E, Budovsky A, Barardo D, Craig T, DIana E, Lehmann G, Toren D, Wang J, Fraifeld VE, De Magalhães JP. Human ageing genomic resources: new and updated databases. Nucleic Acids Res. 2018;46(D1):1083–1090. 10.1093/nar/gkx1042. 10.1093/nar/gkx1042 PMC5753192 29121237

[57] Craig T, Smelick C, Tacutu R, Wuttke D, Wood SH, Stanley H, Janssens G, Savitskaya E, Moskalev A, Arking R, De Magalhães JP (2015). The digital ageing atlas: integrating the diversity of age-related changes into a unified resource. Nucleic Acids Res.

[58] Zierer J, Menni C, Kastenmüller G, Spector TD (2015). Integration of “omics” data in aging research: from biomarkers to systems biology. Aging Cell.

[59] Kuehne A, Hildebrand J, Soehle J, Wenck H, Terstegen L, Gallinat S, Knott A, Winnefeld M, Zamboni N (2017). An integrative metabolomics and transcriptomics study to identify metabolic alterations in aged skin of humans in vivo. BMC Genom.

[60] López-Otín C, Blasco MA, Partridge L, Serrano M, Kroemer G (2013). The hallmarks of aging. Cell.

[61] de Magalhães JP, Curado J, Church GM (2009). Meta-analysis of age-related gene expression profiles identifies common signatures of aging. Bioinformatics.

[62] Dong J, Horvath S (2007). Understanding network concepts in modules. BMC Syst Biol.

[63] Anglani R, Creanza TM, Liuzzi VC, Piepoli A, Panza A, Andriulli A, Ancona N (2014). Loss of connectivity in cancer co-expression networks. PLoS ONE.

[64] Bormann F, Rodríguez-Paredes M, Hagemann S, Manchanda H, Kristof B, Gutekunst J, Raddatz G, Haas R, Terstegen L, Wenck H, Kaderali L, Winnefeld M, Lyko F (2016). Reduced DNA methylation patterning and transcriptional connectivity define human skin aging. Aging Cell.

[65] Southworth LK, Owen AB, Kim SK (2009). Aging mice show a decreasing correlation of gene expression within genetic modules. PLoS Genet.

[66] El Assar M, Angulo J, Rodríguez-Mañas L (2013). Oxidative stress and vascular inflammation in aging. Free Radic Biol Med.

[67] Gopinath SD, Rando TA (2008). Stem cell review series: aging of the skeletal muscle stem cell niche. Aging Cell.

[68] Mann CJ, Perdiguero E, Kharraz Y, Aguilar S, Pessina P, Serrano AL, Muñoz-Cánoves P (2011). Aberrant repair and fibrosis development in skeletal muscle. Skelet Muscle.

[69] Gligorijević N, Zámorová Križáková M, Penezić A, Katrlík J, Nedić O (2018). Structural and functional changes of fibrinogen due to aging. Int J Biol Macromol.

[70] Gysi DM, Voigt A, Fragoso TDM, Almaas E, Nowick K (2018). wTO: an R package for computing weighted topological overlap and a consensus network with integrated visualization tool. BMC Bioinform.

[71] Russo PST, Ferreira GR, Cardozo LE, Bürger MC, Arias-Carrasco R, Maruyama SR, Hirata TDC, Lima DS, Passos FM, Fukutani KF, Lever M, Silva JS, Maracaja-Coutinho V, Nakaya HI (2018). CEMiTool: a Bioconductor package for performing comprehensive modular co-expression analyses. BMC Bioinform.

[72] Tesson BM, Breitling R, Jansen RC (2010). DiffCoEx: a simple and sensitive method to find differentially coexpressed gene modules. BMC Bioinform.

[73] Gysi DM, de Miranda Fragoso T, Zebardast F, Bertoli W, Busskamp V, Almaas E, Nowick K (2020). Whole transcriptomic network analysis using co-expression Differential Network Analysis (CoDiNA). PLoS ONE.

[74] Watson M (2006). CoXpress: differential co-expression in gene expression data. BMC Bioinform.

[75] Hu Z (2014). Using VisANT to analyze networks. Curr Protoc Bioinform.

[76] Shannon P, Markiel A, Ozier O, Baliga NS, Wang JT, Ramage D, Amin N, Schwikowski B, Ideker T (2003). Cytoscape: a software environment for integrated models. Genome Res.

