# Supplementary material for: GWENA: gene co-expression networks analysis and extended modules characterization in a single Bioconductor package
Source: BMC Bioinformatics. 2021 May 25;22:267. doi: 10.1186/s12859-021-04179-4 (PMC8152313; doi:10.1186/s12859-021-04179-4)
Supplement: Supplementary file 1 — Additional file 1. Supplementary Figure 4: Distribution of the connectivity for each gene by module between the two age range. Genes connectivity is ordered by increasing connectivity in the young condition (red). [file 12859_2021_4179_MOESM1_ESM.pdf]

Additional file 1

GWENA: gene co-expression networks analysis and extended modules characterization in a single Bioconductor package

Gwenaëlle G. Lemoine, Marie-Pier Scott-Boyer, Bathilde Ambroise, Olivier Périn, Arnaud Droit

Supplementary Material and Method

1 Z summary detail and combination with NetRep

As NetRep uses a permutation test with the null hypothesis of the module being not preserved, it can only return if the module is preserved or not significant. To determine if a module is not preserved or moderately preserved, a Z summary statistic is computed using the topological metrics defined by Langfelder et al. [1] and renamed by NetRep [2] such as :

$$Z_{summary} = \frac{Z_{density} + Z_{connectivity}}{2}$$

With the NetRep notation :

$$Z_{density} = median(Z_{cor.density}, Z_{avg.edge.wei}, Z_{mod.coh}, Z_{avg.node.contrib})$$
$$Z_{connectivity} = median(Z_{concor.wei.deg}, Z_{concor.nod.contrib}, Z_{concor.cor})$$

Where :

$$cor.density = \text{mean}(vect.Matrix(\text{sign}(r_{ij}^{[ref](q)} rij^{[test](q)})))$$
$$avg.edge.wei = density^{[test](q)} = \text{mean}(vect.Mat(A^{[test](q)}))$$
$$mod.coh = \text{mean}_{i \in Mq}((kME_i^{[test](q)})^2)$$
$$avg.node.contrib = \text{mean}_{i \in Mq}(\text{sign}(kME_i^{[ref](q)})kME_i^{[test](q)})$$
$$concor.wei.deg = \text{cor}(kIM)^{[ref](q)}, kIM^{[test](q)}$$
$$concor.nod.contrib = \text{cor}_{i \in Mq}(kME_i^{[ref](q)}, kME_i^{[test](q)})$$
$$concor.cor = \text{cor}(vect.Matrix(r^{[ref](q)}), vect.Matrix(r^{[test](q)}))$$

$$vect.Matrix(A) = (a_{2,1}, a_{3,1}, ..., a_{n,1}, a_{n,n-1})$$
$$r_{ij}^{[ref]} = \text{cor}(x_i^{[ref]}, x_j^{[ref]})$$
$$r_{ij}^{[test]} = \text{cor}(x_i^{[test]}, x_j^{[test]})$$

This score returns :

- **Preserved** if the $Z_{summary}$ is above 10
- **Moderately preserved** if the $Z_{summary}$ is between 2 and 10
- **Unpreserved** if the $Z_{summary}$ is below 2

The results from both NetRep permutation test and the $Z_{summary}$ are then combined in GWENA as shown in Figure 1 and return a final result on the module comparison.

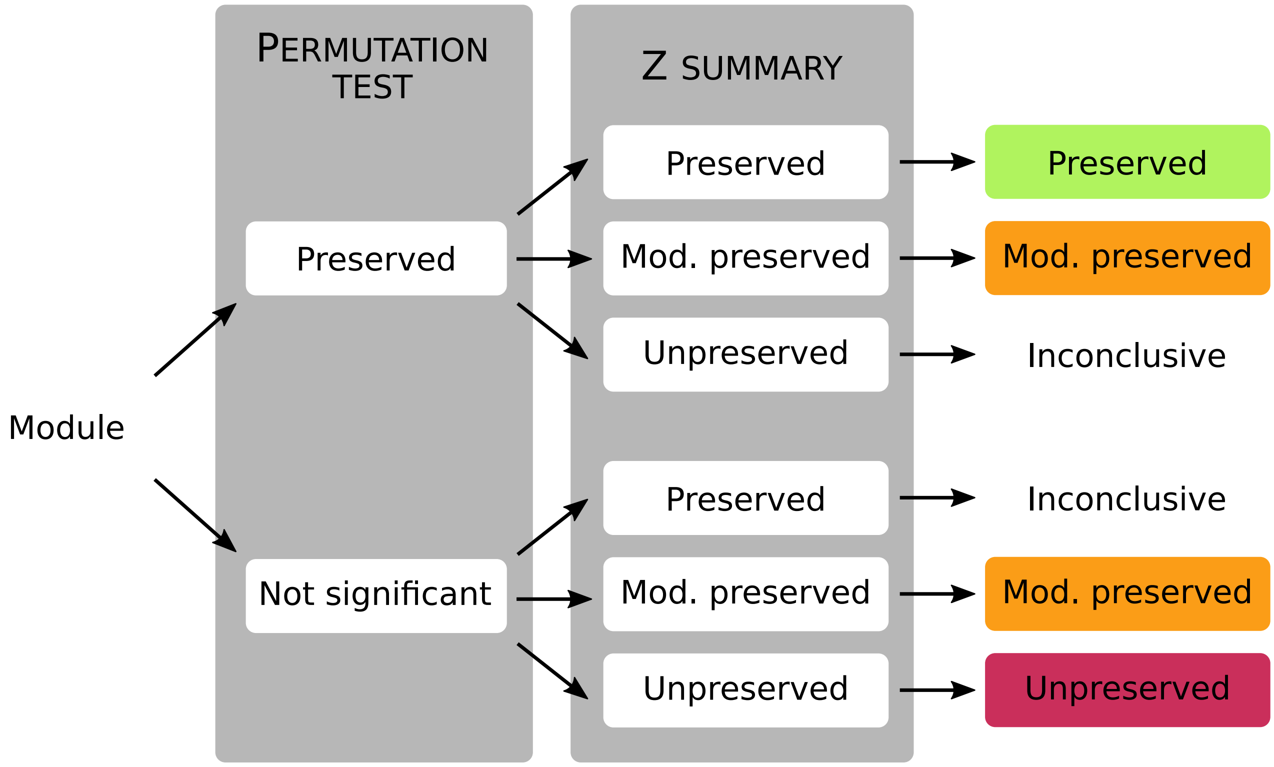

Figure 1: Combination of the permutation test result ans the $Z_{summary}$ result in GWENA to return a final result on the module comparison.

2 Details on case study data

Public data were obtained from GTEx v8 version on the GTEx Portal on 09/20/2020. Access to private data was subject to a request to dbGaP on accession number phs000424.v8.p2. Data were obtained on 10/21/2020.

Data	File
Gene expression	GTEx_Analysis_2017-06-05_v8_RNASeQCv1.1.9_gene_reads.gct.gz
Public annotation	GTEx_Analysis_v8_Annotations_SampleAttributesDS.txt
Private annotation	phs000424.v8.pht002742.v8.p2.c1.GTEx_Subject_Phenotypes.GRU.txt
Phenotype	GTEx_Analysis_v8_Annotations_SubjectPhenotypesDS.txt

Table 1: Correspondence between file names and their contents

3 GTEx data normalization with PC-correction method

In order to limit batch effect and handle the maximum of other co-founding effects, we chose to use a method based on PC-correction as recommended by Parsana et al. [3] for GTEx data. However age is usually included in this confounding factors, therefore is corrected. Since we're interested in gene changes we adapted the method to remove only the top n PC correlated to age and which removed the least of genes correlating with age. The n number of PC to remove was estimated by calculating the loss of correlation between phenotype and genes expression (Figure 2) and confirmed by looking for the number of significantly correlated genes with two ageing gene databases (Figure 3): GenAge [4] and Digital Aging Atlas [5].

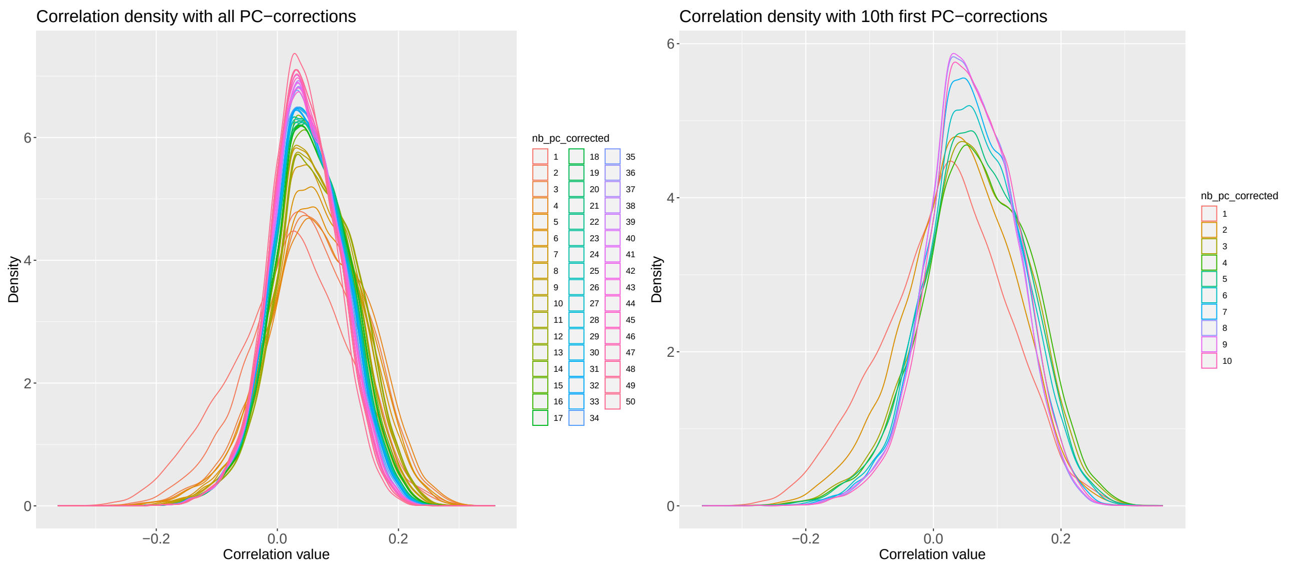

Figure 2: Ageing genes correlation density with phenotype depending on the number of PC corrected. Left figure contains all PC correction tested. For clarity we filtered on the first 10 PC corrected on the right figure.

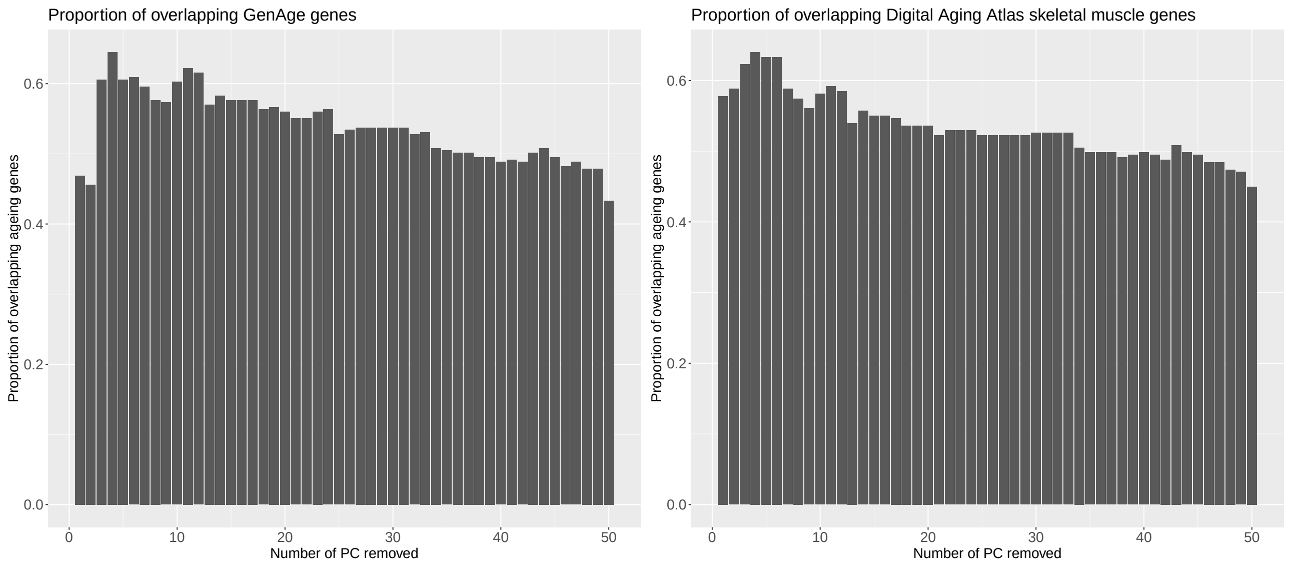

Figure 3: Number of genes known to be associated with ageing.

Correlation density in Figure 2 suggest a similarity between the corrections from 2 to 5 PC removal. Combined with the proportion of overlapping known ageing genes in Figure 3 we determined the optimal number of PC n to remove to be 4.

Supplementary Results

4 Connectivity drop on all modules

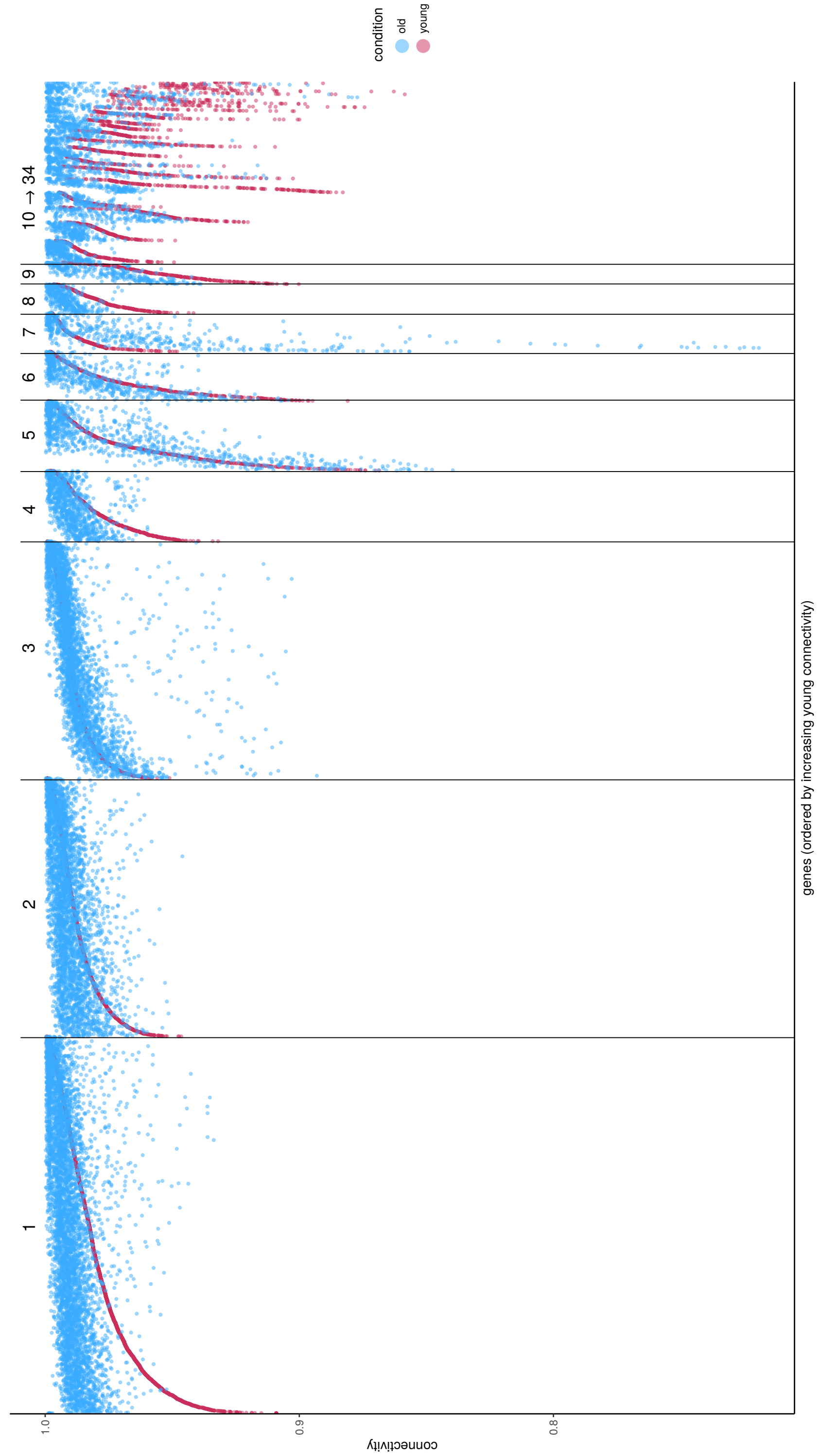

Figure 4: Here is a caption of the figure which is so long that it has to be wrapped over multiple lines, but should not exceed the width (height after the rotation) of the image.

5 New enrichment terms in sub module 6 from module 7 old age range

Table 2: Enrichment table from module 7 sub module 6 in old condition. Terms are sorted along their novelty (is the enrichment new compared to the enrichments from sub modules in the young age range) and then the source. Source is the enrichment database used on the gene set (GO:BP = Gene Ontology : Biological Process, GO:CC = Gene Ontology Cellular Compartment, GO:MF = Gene Ontology : Molecular Function, HP : Human Phenotype Ontology, WP = WikiPathway, KEGG = Kyoto Encyclopedia of Genes and Genomes, REAC = Reactome, TF = Transfac).

source	term name	is new	source	term name	is new
CORUM	Fibrinogen complex	no	GO:BP	regulation of vasoconstriction	yes
GO:BP	fibrinolysis	no	GO:BP	heterotypic cell-cell adhesion	yes
GO:BP	negative regulation of blood coagulation	no	GO:BP	platelet aggregation	yes
GO:BP	negative regulation of hemostasis	no	GO:BP	regulation of endothelial cell apoptotic process	yes
GO:BP	negative regulation of coagulation	no	GO:BP	endothelial cell apoptotic process	yes
GO:BP	negative regulation of wound healing	no	GO:BP	protein processing	yes
GO:BP	negative regulation of response to wounding	no	GO:BP	cell-matrix adhesion	yes
GO:BP	regulation of body fluid levels	no	GO:BP	positive regulation of response to wounding	yes
GO:CC	blood microparticle	no	GO:BP	positive regulation of blood circulation	yes
GO:CC	platelet alpha granule lumen	no	GO:BP	vasoconstriction	yes
GO:CC	platelet alpha granule	no	GO:BP	regulation of vesicle-mediated transport	yes
GO:CC	collagen-containing extracellular matrix	no	GO:BP	cell adhesion	yes
GO:CC	fibrinogen complex	no	GO:BP	biological adhesion	yes
GO:CC	extracellular space	no	GO:BP	homotypic cell-cell adhesion	yes
GO:CC	extracellular exosome	no	GO:BP	vascular process in circulatory system	yes
GO:CC	extracellular vesicle	no	GO:BP	extrinsic apoptotic signaling pathway via death domain receptors	yes
GO:CC	extracellular organelle	no	GO:CC	secretory granule lumen	yes
GO:CC	extracellular region	no	GO:CC	cytoplasmic vesicle lumen	yes
GO:CC	secretory granule	no	GO:CC	vesicle lumen	yes
GO:CC	secretory vesicle	no	GO:CC	extracellular matrix	yes
GO:CC	vesicle	no	GO:CC	cell surface	yes
GO:MF	enzyme inhibitor activity	no	GO:CC	endoplasmic reticulum lumen	yes
HP	Splenic rupture	no	GO:CC	cytoplasmic vesicle	yes
WP	COVID-19, thrombosis and anticoagulation	no	GO:CC	intracellular vesicle	yes
GO:BP	platelet degranulation	yes	GO:CC	chylomicron	yes
GO:BP	regulation of blood coagulation	yes	GO:CC	very-low-density lipoprotein particle	yes
GO:BP	regulation of hemostasis	yes	GO:CC	triglyceride-rich plasma lipoprotein particle	yes
GO:BP	regulation of coagulation	yes	GO:CC	external side of plasma membrane	yes
GO:BP	regulation of wound healing	yes	GO:CC	high-density lipoprotein particle	yes
GO:BP	plasminogen activation	yes	GO:CC	plasma lipoprotein particle	yes
GO:BP	regulation of response to wounding	yes	GO:CC	lipoprotein particle	yes
GO:BP	protein activation cascade	yes	GO:CC	protein-lipid complex	yes
GO:BP	blood coagulation, fibrin clot formation	yes	GO:MF	signaling receptor binding	yes
GO:BP	vesicle-mediated transport	yes	GO:MF	chaperone binding	yes
GO:BP	regulated exocytosis	yes	GO:MF	immunoglobulin binding	yes
GO:BP	negative regulation of fibrinolysis	yes	GO:MF	lipoprotein particle receptor binding	yes

GO:BP	exocytosis	yes
GO:BP	zymogen activation	yes
GO:BP	blood coagulation	yes
GO:BP	hemostasis	yes
GO:BP	coagulation	yes
GO:BP	regulation of fibrinolysis	yes
GO:BP	positive regulation of heterotypic cell-cell adhesion	yes
GO:BP	regulation of cell-substrate adhesion	yes
GO:BP	negative regulation of response to external stimulus	yes
GO:BP	negative regulation of blood vessel diameter	yes
GO:BP	negative regulation of response to stimulus	yes
GO:BP	regulation of heterotypic cell-cell adhesion	yes
GO:BP	positive regulation of blood coagulation	yes
GO:BP	positive regulation of hemostasis	yes
GO:BP	positive regulation of coagulation	yes
GO:BP	wound healing	yes
GO:BP	negative regulation of multicellular organismal process	yes
GO:BP	positive regulation of cell-substrate adhesion	yes
GO:BP	secretion by cell	yes
GO:BP	negative regulation of endothelial cell apoptotic process	yes
GO:BP	positive regulation of vasoconstriction	yes
GO:BP	export from cell	yes
GO:BP	negative regulation of cellular process	yes
GO:BP	regulation of response to stress	yes
GO:BP	positive regulation of substrate adhesion-dependent cell spreading	yes
GO:BP	regulation of blood vessel diameter	yes
GO:BP	regulation of tube diameter	yes
GO:BP	negative regulation of extrinsic apoptotic signaling pathway via death domain receptors	yes
GO:BP	regulation of tube size	yes
GO:BP	cell-substrate adhesion	yes
GO:BP	post-translational protein modification	yes
GO:BP	response to wounding	yes
GO:BP	secretion	yes
GO:MF	extracellular matrix structural constituent	yes
HP	Menometrorrhagia	yes
HP	Abnormality of the common coagulation pathway	yes
HP	Spontaneous abortion	yes
HP	Abnormality of coagulation	yes
HP	Hypofibrinogenemia	yes
HP	Abnormality of circulating fibrinogen	yes
HP	Joint swelling	yes
HP	Abnormality of the coagulation cascade	yes
HP	Abnormal delivery	yes
HP	Abnormal thrombosis	yes
KEGG	Complement and coagulation cascades	yes
KEGG	Platelet activation	yes
KEGG	Cholesterol metabolism	yes
MIRNA	hsa-miR-409-3p	yes
MIRNA	hsa-miR-144-3p	yes
REAC	Platelet degranulation	yes
REAC	Response to elevated platelet cytosolic Ca2+	yes
REAC	Platelet activation, signaling and aggregation	yes
REAC	Regulation of Insulin-like Growth Factor (IGF) transport and uptake by Insulin-like Growth Factor Binding Proteins (IGFBPs)	yes
REAC	Post-translational protein phosphorylation	yes
REAC	Hemostasis	yes
REAC	GRB2:SOS provides linkage to MAPK signaling for Integrins	yes
REAC	p130Cas linkage to MAPK signaling for integrins	yes
REAC	Regulation of TLR by endogenous ligand	yes
REAC	Common Pathway of Fibrin Clot Formation	yes
REAC	Integrin signaling	yes
REAC	Signaling by high-kinase activity BRAF mutants	yes
REAC	Platelet Aggregation (Plug Formation)	yes
REAC	Formation of Fibrin Clot (Clotting Cascade)	yes
REAC	MAP2K and MAPK activation	yes
REAC	Signaling by moderate kinase activity BRAF mutants	yes
REAC	Signaling downstream of RAS mutants	yes

GO:BP	regulation of response to external stimulus	yes	REAC	Paradoxical activation of RAF signaling by kinase inactive BRAF	yes
GO:BP	platelet activation	yes	REAC	Signaling by RAS mutants	yes
GO:BP	transport	yes	REAC	Signaling by BRAF and RAF fusions	yes
GO:BP	response to stress	yes	REAC	Oncogenic MAPK signaling	yes
GO:BP	establishment of localization	yes	REAC	Dissolution of Fibrin Clot	yes
GO:BP	negative regulation of epithelial cell apoptotic process	yes	REAC	Integrin cell surface interactions	yes
GO:BP	regulation of cell adhesion	yes	TF	Factor: HNF1A; motif: GGTTAATNATTAMC	yes
GO:BP	regulation of substrate adhesion-dependent cell spreading	yes	TF	Factor: HNF-1alpha; motif: GGTTAATNWTTAMCN	yes
GO:BP	induction of bacterial agglutination	yes	TF	Factor: Sox-2; motif: NNNNNAACAAWGN; match class: 1	yes
GO:BP	regulation of response to stimulus	yes	WP	Folate Metabolism	yes
GO:BP	proteolysis	yes	WP	Selenium Micronutrient Network	yes
GO:BP	negative regulation of biological process	yes	WP	Human Complement System	yes
GO:BP	regulation of cell-cell adhesion	yes	WP	Blood Clotting Cascade	yes
GO:BP	regulation of extrinsic apoptotic signaling pathway via death domain receptors	yes	WP	Fibrin Complement Receptor 3 Signaling Pathway	yes
GO:BP	positive regulation of wound healing	yes	WP	Vitamin B12 Metabolism	yes

The distribution of the newly and previously found terms in the enrichment analysis across the the sub-modules from young and old age range (Figure 5).

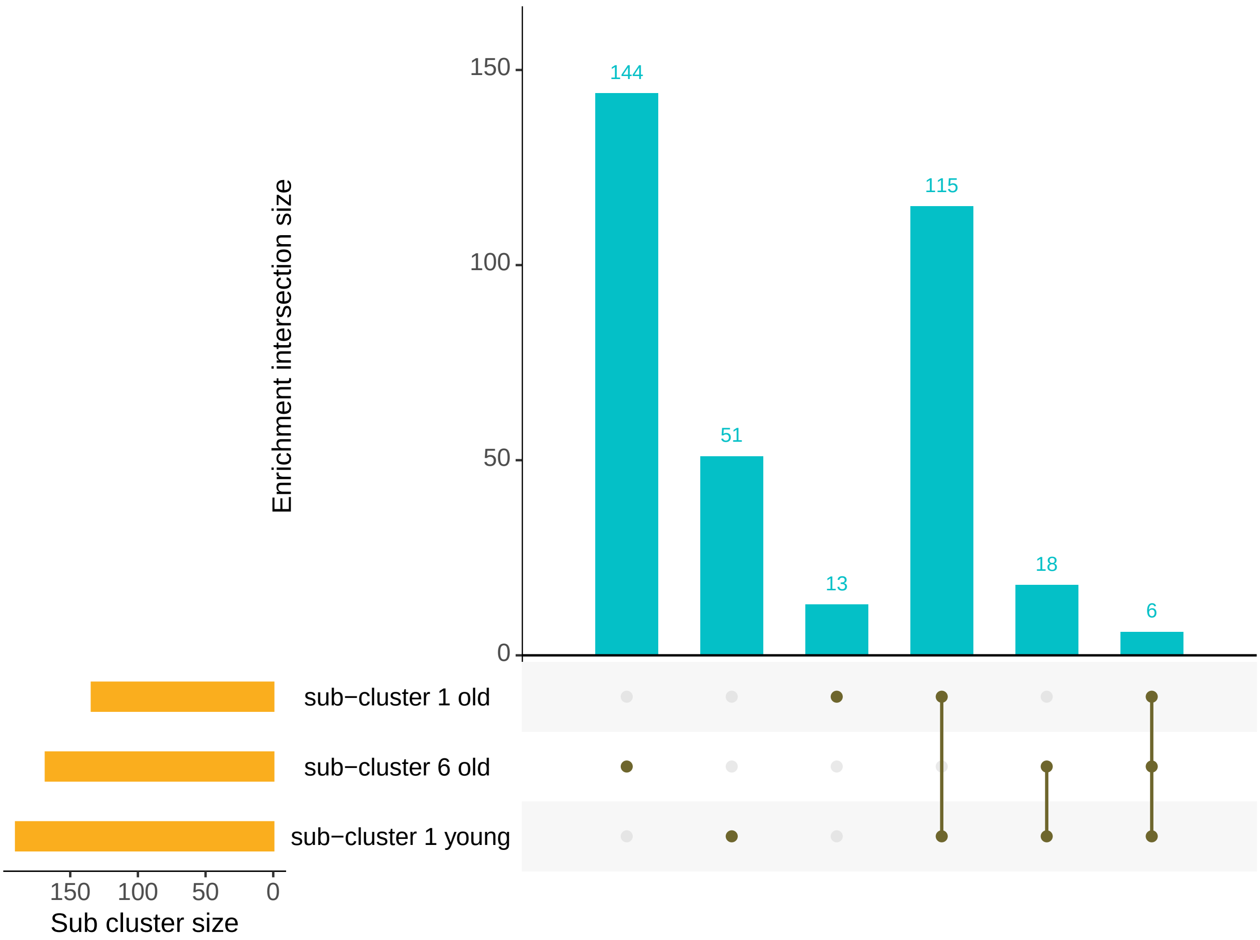

Figure 5: Overlap between the enrichments found in sub-cluster 1 young, sub-cluster 1 old, and sub-cluster 6 old.(Upset diagram)

References

[1] Peter Langfelder, Rui Luo, Michael C. Oldham, and Steve Horvath. Is my network module preserved and reproducible? *PLoS Computational Biology*, 7(1), 2011.

[2] Scott C. Ritchie, Stephen Watts, Liam G. Fearnley, Kathryn E. Holt, Gad Abraham, and Michael Inouye. A Scalable Permutation Approach Reveals Replication and Preservation Patterns of Network Modules in Large Datasets. *Cell Systems*, 3(1):71–82, 2016.

[3] Princy Parsana, Claire Ruberman, Andrew E. Jaffe, Michael C. Schatz, Alexis Battle, and Jeffrey T. Leek. Addressing confounding artifacts in reconstruction of gene co-expression networks. *Genome Biology*, 20(1):94, 2019.

[4] Robi Tacutu, Daniel Thornton, Emily Johnson, Arie Budovsky, DIogo Barardo, Thomas Craig, Eugene DIana, Gilad Lehmann, Dmitri Toren, Jingwei Wang, Vadim E. Fraifeld, and Joaõ P. De Magalhães. Human Ageing Genomic Resources: New and updated databases. *Nucleic Acids Research*, 46(D1):D1083–D1090, 2018.

[5] Thomas Craig, Chris Smelick, Robi Tacutu, Daniel Wuttke, Shona H. Wood, Henry Stanley, Georges Janssens, Ekaterina Savitskaya, Alexey Moskalev, Robert Arking, and João Pedro De Magalhães. The Digital Ageing Atlas: Integrating the diversity of age-related changes into a unified resource. *Nucleic Acids Research*, 43(D1):D873–D878, 2015.
